# Membranoproliferative glomerulonephritis recurrence after kidney transplantation: using the new classification

**DOI:** 10.1186/s12882-015-0219-x

**Published:** 2016-01-11

**Authors:** Sami Alasfar, Naima Carter-Monroe, Avi Z. Rosenberg, Robert A. Montgomery, Nada Alachkar

**Affiliations:** Department of Medicine, The Johns Hopkins University School of Medicine, 600 Wolfe Street. Brady 502, 21287 Baltimore, MD USA; Department of Pathology, The Johns Hopkins University School of Medicine, 600 N Wolfe St, Baltimore, MD 21287 USA; Department of Pathology, Children’s National Medical Center, 111 Michigan Ave. NW, Washington, DC 20010 USA; Department of Surgery, The Johns Hopkins University School of Medicine, 600 N Wolfe St, Baltimore, MD 21287 USA

**Keywords:** MPGN, Kidney transplant, C3 glomerulopathy, Immune complex GN

## Abstract

**Background:**

Membranoproliferative glomerulonephritis (MPGN) is an uncommon glomerular disorder that may lead to end stage renal disease (ESRD). With new understanding of the disease pathogenesis, the classical classification as MPGN types I, II, III has changed. Data on post-transplant MPGN, in particular with the newly refined classification, is limited. We present our center’s experience of MPGN after kidney transplantation using the new classification.

**Methods:**

This is a retrospective study of 34 patients with ESRD due to MPGN who received 40 kidney transplants between 1994 and 2014. We reviewed the available biopsies’ data using the new classification. We assessed post transplantation recurrence rate, risk factors of recurrence, the response to therapy and allografts’ survival.

**Results:**

Median time of follow up was 5.3 years (range 0.5–14 years). Using the new classification, we found that pre-transplant MPGN disease was due to immune complex-mediated glomerulonephritis (ICGN) in 89 % of cases and complement-mediated glomerulonephritis (CGN) in 11 %. Recurrence was detected in 18 transplants (45 %). Living related allografts (*P* = 0.045), preemptive transplantations (*P* = 0.018), low complement level (*P* = 0.006), and the presence of monoclonal gammopathy (*P* = 0.010) were associated with higher recurrence rate in ICGN cases. Half of the patients with recurrence lost their allografts. The use of ACEi/ARB was associated with a trend toward less allograft loss.

**Conclusions:**

MPGN recurs at a high rate after kidney transplantation. The risk of MPGN recurrence increases with preemptive transplantation, living related donation, low complement level, and the presence of monoclonal gammopathy. Recurrence of MPGN leads to allograft failure in half of the cases.

## Background

Membranoproliferative glomerulonephritis (MPGN) is an uncommon glomerular injury pattern characterized by mesangial hypercellularity, endocapillary proliferation, and capillary-wall remodeling. MPGN accounts for approximately 7 to 10 % of all cases of biopsy-confirmed glomerulonephritis [1, 2]. The clinical presentation is variable depending on the pathogenesis involved and the timing of biopsy, and could range from asymptomatic hematuria and/or proteinuria to rapidly progressive glomerulonephritis [3]. Standardized optimal treatment of MPGN is not determined. Commonly used therapies include: glucocorticoids, azathioprine, mycophenolate mofetil, cyclophosphamide, rituximab, plasmapheresis, and eculizumab. There are no randomized controlled trials that assess the effectiveness of individual interventions due in part to the rarity of MPGN. In up to 50 % of the cases, MPGN may take a progressive course and lead to end stage renal disease (ESRD) within 8–10 years of presentation [4].

Historically, MPGN has been classified into three types based on the location and the appearance of immune deposits under observed electron microscopy (EM). MPGN type I is by far the most common form and characterized by subendothelial and mesangial deposits. MPGN type II, also known dense deposits disease, is characterized by highly osmiophilic dense deposits within the lamina densa of the glomerular basement membrane. MPGN type III is characterized by subepithelial and subendothelial deposits [5]. Certain systemic diseases such as chronic hepatitis C, autoimmune diseases, and plasma cell dyscrasias, have been associated with MPGN and in these cases it is called secondary MPGN and by histomorphology may present as MPGN types I or III [6].

Recent advances in understanding the pathophysiology of MPGN and the role of the alternative complement pathway have led to the development of a new classification system of MPGN types. This classification is based on the mechanism involved in the glomerular injury rather than the location of and appearance of deposits. This may help to direct the clinical evaluation and provide more pathophysiology-specific treatments. It has been known that the classic glomerular injuries seen in MPGN are the results of the deposition of immunoglobulins, complement factors, or both in the glomerular mesangium and along the glomerular capillary walls. These can be distinguished by immunofluorescence microscopy. Therefore, MPGN can be viewed as either immune-complex-mediated glomerulonephritis (ICGN) which is characterized by the presence of immune complexes and complement components, or complement-mediated (CGN) which is characterized by the presence of complement components in the absence of immune complexes.

Several observational studies assessed MPGN recurrence after kidney transplantation, however, the data on the natural course of MPGN recurrence, impact on renal allograft, and its treatment remains limited due to the low number of patients and short follow-up time in these studies. In addition, these studies assessed MPGN recurrence based on the old classification system. MPGN often recurs after kidney transplantation and the reported rate of recurrence of MPGN is quite variable (19–65 %) depending on the study [7–9]. What was formerly called MPGN type II was found to be associated with the highest rate of recurrence after transplantation [10, 11]. Recurrence after transplantation was found to be associated with the presence of monoclonal immunoglobulins [8], lower serum complement level [8], higher proteinuria [11], human leukocyte antigen (HLA) B8, DR3 [9], B49, and DR4 [7], and the presence of crescents in the original biopsy [11].

We, herein, share our center’s experience of kidney transplantation course in patients with ESRD due to MPGN. We assessed several possible risk factors as well as recurrence rate, the impact of the recurrence on renal allografts, and the response to treatment; all in light of the new MPGN classification.

## Methods

### Study design

This is a 20-year retrospective study of all patients with MPGN who received kidney transplantation in our center. The study was approved by the Johns Hopkins Medicine Institutional Review Board. We identified 34 patients with ESRD due to MPGN who received 40 total kidney transplantations in our institution between January 1994 and September 2014. We confirmed the diagnosis by reviewing all the available biopsy data in the patients’ medical records, in addition to a second review of the biopsies by our pathologist for the purpose of this study. The other purpose of the second review of all the biopsies was to utilize the new classification of the MPGN types. We aimed to assess the clinical outcome of the patients and allografts using the new classification of MPGN. We also identified the risk factors of recurrence, response to therapy and long-term prognosis. After we confirmed the diagnosis of MPGN of our cohort, we reviewed all available medical records and we collected the pertinent clinical data.

### Clinical data

Five patients had repeated transplants. One patient had 3 transplants; the first failed due to vascular thrombosis, the second failed due to MPGN recurrence and rejection, and the third failed due to severe acute tubular injury (ATN). The second patient had 2 transplants; the first failed due to ATN, and the second did not fail but it had MPGN recurrence. The third patient had 2 transplants and both failed due to MPGN recurrence. The fourth patient had 2 transplants, the first failed due to rejection (with no recurrence) and the second did not fail and had no recurrence. The fifth patient had 2 transplants; both failed due to rejection and MPGN recurrence.

### Statistical analysis

We performed our statistical analyses using STATA 13 statistical software (StataCorp LP, College Station, Texas, USA). Descriptive statistics were used to estimate the frequencies, means, medians, and proportions of the study variables. We checked normality of distribution for continuous variables using box plots, normal probability plots, and Shapiro/Wilk normality test. Continuous data were expressed as median and range or mean and standard deviation. We used survival analysis/Kaplan-Meier curve to present all allograft survivals. We used Cox regression models to compare between the recurrence and non-recurrence groups. A *P*-value of <0.05 is considered statistically significant.

## Results

Patients’ characteristics are shown in Table 1. Mean age at transplantation was 37.4 ± 13.5 years. Half of the transplants were done in men. Twenty-eight (70 %) out of the allografts were done in Caucasians and seven (17 %) were done in African-American. Out of these 40 transplantations, 4 (10 %) were preemptive. Living-related donors kidney transplants were performed in 15 of all transplants, additional 15 were from living unrelated donors, and the remainders were from deceased donors. All cases were biopsy confirmed MPGN; 65 % were diagnosed as MPGN type I, 9 % were MPGN type II, 21 % were MPGN type III, and 5 % had features of both MPGN type II and III. Upon reclassification using the new classification system, 88 % were ICGN and 12 % were CGN. None of the cases were dense deposits disease.

**Table 1:** Transplants’ Characteristics

Variable	Transplants (*n* = 40)
Median age at transplantation- yr (range)	37.4 (15–59)
Gender (Male)	20 (50 %)
Race: Caucasian	28 (70 %)
African American	7 (17 %)
Other	5 (13 %)
MPGN type (Old classification^a^): Type I	65 %
Type II	9 %
Type III	21 %
Mixed	5 %
MPGN type (New classification^b^): ICGN	88 %
CGN	12 %
Donor source: Deceased	10 (25 %)
Living unrelated	15 (37 %)
Living related	15 (37 %)
Number of mismatches	
0	1 (2 %)
1	3 (7 %)
2	5 (12 %)
3	10 (25 %)
4	7 (17 %)
5	7 (17 %)
6	6 (15 %)
Preemptive kidney transplant	4 (10 %)
Median cumulative ESRD duration for non-preemptive- yr (range)	5.2 (0.2–20)

^a^Old classification is based on location and appearance of immune deposits under electron microscopy

^b^New classification is based on C3 and IgG staining with immunofluorescence

Most of the cases were classified as idiopathic MPGN. In regards to secondary causes of MPGN, hepatitis C (HCV) antibodies and positive serum HCV PCR were positive in only one transplant. Fifteen of the total 40 transplants had cryoglobulin checked and it was negative in all of them. None of the patients had evidence of other autoimmune disease such as positive ANA, anti dsDNA, rheumatoid factor, or anti SSA/SSB antibodies. None had a diagnosis of malignancy prior to transplant. In regards to monoclonal gammopathy and/or myeloma as a cause of MPGN, we identified 19 patients who had serum and/or urine electrophoresis checked and only 7 of them had evidence of a monoclonal spike. None of the patients had a diagnosis of frank multiple myeloma prior to transplant but one patient, who also had a monoclonal spike prior to transplant, developed multiple myeloma after transplant. Based on the data we have we could not tell whether some of the cases were secondary to a systemic infection. Cumulative mean ESRD duration prior to transplant in non-preemptive transplants was 5.2 (0.25–20) years. Only 2 out of the 40 transplants were ABO incompatible. Induction immunosuppression was thymoglobulin in 23, daclizumab in 15, and basiliximab in 2 of the transplantations. All cases, except one, received calcineurin inhibitor- based maintenance immunosuppression; one received mammalian target of rapamycine (mTOR inhibitor)- based therapy. Five out of the 40 transplants had delayed graft function (DGF) after transplantation. Seven cases were placed on angiotensin-converting enzyme (ACE) inhibitors or angiotensin receptor blockers (ARB) after kidney transplantation. Median duration of follow up period is 5.3 years (range 0.5–14 years). Two patients died during the follow up period following graft loss and the cause of death is not available to us. Seventeen of the forty renal allografts (42 %) failed due to different reasons other than MPGN recurrence. Causes of graft loss are shown in Table 2.

**Table 2:** Reasons for renal allografts loss

Reason for graft loss	Frequency
MPGN recurrence	6
Antibody-Medicated Rejection	2
Cell-Medicated rejection	2
MPGN recurrence & rejection^a^	3
ATN	2
Bleeding	1
Thrombosis	1

^a^In the three cases recurrence preceded rejection and the rejection was antibody mediated

### MPGN recurrence

Post-transplant recurrent MPGN was detected in 18 out of the 40 transplants (45 %). When clinically indicated, diagnosis was made by renal allograft biopsies. Indications for obtaining renal allograft biopsy in these patients included: decreased estimated glomerular filtration rate (eGFR) (10 allografts), proteinuria (4 allografts), decreased eGFR and proteinuria (3 allografts), and protocol post-transplant biopsy (1 allografts). Ten out of the 18 (55 %) recurrences were diagnosed within the first year post-transplant. Median time to recurrence was 8 months (range 1–108 months). Table 3 shows the characteristics of patients who developed post-transplant MPGN recurrence.

**Table 3:** Shows characteristics of allografts with post-transplant MPGN recurrence

Graft	MPGN type (Native/Rec)		Age at Txp	Sex/Race	Donor	Months to recurrence	sCr	Proteinuria(g)	Rx	Graft status/sCr
	By EM	By IF								
1	1/1	IC/IC	20	F/AA	LR	2	1.4	4	None	Functional/1.6
2	1 ^a^/1	IC/IC	39	F/C	LR	3	1.2	9	CS + ACEi	Functional/ 2.5
3	1 ^a^/1	C3/IC	59	F/C	LR	1	0.9	2.7	None	Functional/0.9
4	1/1	IC/IC	26	M/C	LR	24	2	2.3	Ritux + CS	Lost in 22 mos
5	2 + 3/2 + 3	IC/IC	53	M/C	D	24	3	NA	CS	Lost in 96 mos
6	3 ^a^/1	IC/IC	18	M/I	LU	36	2.5	4	TPE + CS	Lost in 4 mos
7	3 ^a^/?	IC/IC	15	M/I	LR	18	NA	NA	None	Lost in 1 mos
8	1/1	IC/IC	28	M/A	LR	6	1.8	0.1	Ritux	Functional/1.2
9	1/1	IC/IC	54	M/AA	D	4	1.0	2	ACEi	Functional/1.0
10	1/1	IC/IC	53	M/C	D	4	1.7	0.5	TPE+ ACEi	Functional/1.2
11	3^a^/1	IC/IC	30	F/C	LR	108	2.0	0.45	Ritux	Functional/2.5
12	2/2	IC/IC	54	M/C	LU	24	3.4	NA	Ritux	Lost in 2 mos
13	1/1	IC/IC	51	F/C	LU	1	1.9	0.8	TPE + Ritux	Functional/1.7^b^
14	1^a^/1	IC/IC	32	M/H	LR	24	1.7	4	TPE + Ritux	Lost in 41 mos
15	1/1	IC/IC	25	F/C	LR	14	1.3	9.5	Ritux	Lost in 4 mos
16	1/1	IC/IC	53	F/AA	LR	12	NA	NA	CS	Lost in 8 mos
17	1/2	IC/C3	58	F/AA	D	6	3.1	NA	None	Lost in 5 mos
18	1/1	C3/C3	24	M/C	LU	3	1.3	0.2	Eculizumab	Functional/1.3

*Txp* transplant, *IC* immune complex, *sCr* serum Creatinine, *Rx* Treatment, *AA* African American, *C* Caucasian, *I* Indian, *A* Asian, *H* Hispanic, *LR* living related, *LU* living unrelated, *D*: deceased, *CS* corticosteroids, *ACEi* Angiotensin Converting Enzyme inhibitor, *TPE* therapeutic plasma exchange, *Ritux* Rituximab, *NA* not available

^a^MPGN type by documentation but kidney biopsy slides are not available for review. All others are classified based on kidney biopsy slides review

^b^Patient diagnosed with plasma cell dyscrasia and received chemotherapy

### Factors associated with MPGN recurrence

Table 4 shows the effect of different variables associated with MPGN recurrence after kidney transplantation by univariate Cox analysis. We only included ICGN patients in analysis because of the small number of CGN patients and the difference in pathophysiology involved.

**Table 4:** Variables associated with MPGN ICGN-type recurrence after kidney transplantation by univariate Cox analysis (allograft *n* = 35)

Independent variable	Hazard ratio (CI)	*P*-value
Age at transplantation	1.019 (0.937–1.018)	0.65
Gender (Male)	1.00 (0.118–8.420)	1
Race (Caucasian)	1.5 (0.120–18.411)	0.1
Allograft source (Living related)	10.19 (0.866–12.96)	0.045
Duration of dialysis	0.951 (0.775–1.167)	0.612
Preemptive transplantation	6.322 (1.455–12.411)	0.018
Previous failed transplantation	0.833 (0.098–7.026)	0.86
DGF (In deceased donor)	1.21 (0.158–9.508)	0.83
Development of rejection	3.148 (0.854–9.546)	0.25
Use of ACEi/ARB	1.312 (0.587–5.847)	0.658
Low complement level	5.522 (1.632–18.679)	0.006
Evidence of monoclonal gammopathy	5.606 (1.522–20.642)	0.010

Only cases with confirmed MPGN type by kidney biopsy review and/or nephrology documentation are included

1. Complement:
  Levels of the serum complement component C3 and/or serum complement component C4 were available in 22 out of the 40 transplants (56 %) in pre-transplant period. Among the ICGN cases, eight of the thirteen (72 %) patients who developed post-transplant recurrent MPGN and had complement levels available, had either low C3 or C4 level. On the other hand, all (100 %) 9 patients who did not develop post-transplant recurrent MPGN and had complement levels available had normal C3 and C4 levels. This difference was statistically significant (*P* = 0.006) with a hazard ratio (HR) of 5.5.
2. Monoclonal gammopathy:
  To assess the frequency and association of plasma cell dyscrasias and MPGN recurrence, we identified a total of 19 patients who had serum or urine protein electrophoresis checked at some point before or after kidney transplantation. Out of the ICGN cases, 6ix of the 10 (60 %) patients, who developed post-transplant recurrent MPGN and had serum or urine electrophoresis available, had monoclonal proteins. On the other hand, only 1 out of the 9 (11 %) patients, who did not develop post-transplant recurrent MPGN and had serum or urine electrophoresis available, had monoclonal protein. This difference was also statistically significant (*P* = 0.01) and with a HR of 5.6.
3. Allograft type
  Among the ICGN cases, when the source of donor was evaluated, living-related kidney transplants were associated with the highest risk of recurrence 8 out of 13 (61 %) compared to the other types (living-unrelated and deceased donor) 87 out of 20 (35 %), *P* = 0.045 and HR of 10.41.
4. Type of MPGN:
  Based on the historical (EM based) classification of MPGN, none of the specific MPGN subtypes were associated with a higher risk of recurrence compared to the other types (HR of 1.901 and *P* = 0.28). After reclassification the original MPGN based on immunofluorescence pattern, we did not observe any association between MPGN class and recurrence after transplantation (HR 0.6 if ICGN and *P* = 0.50).
5. Other factors:

There was no effect for race, gender, age, number of transplants, degree of mismatch, and development of DGF or rejection on risk of recurrence. Duration of dialysis prior to transplant was not associated with increased risk of MPGN recurrence. However, preemptive transplant was associated with increased risk of post-transplant recurrent MPGN (HR of 6.32 and *P* = 0.018).

### MPGN recurrence reclassification

**Fig. 1:**
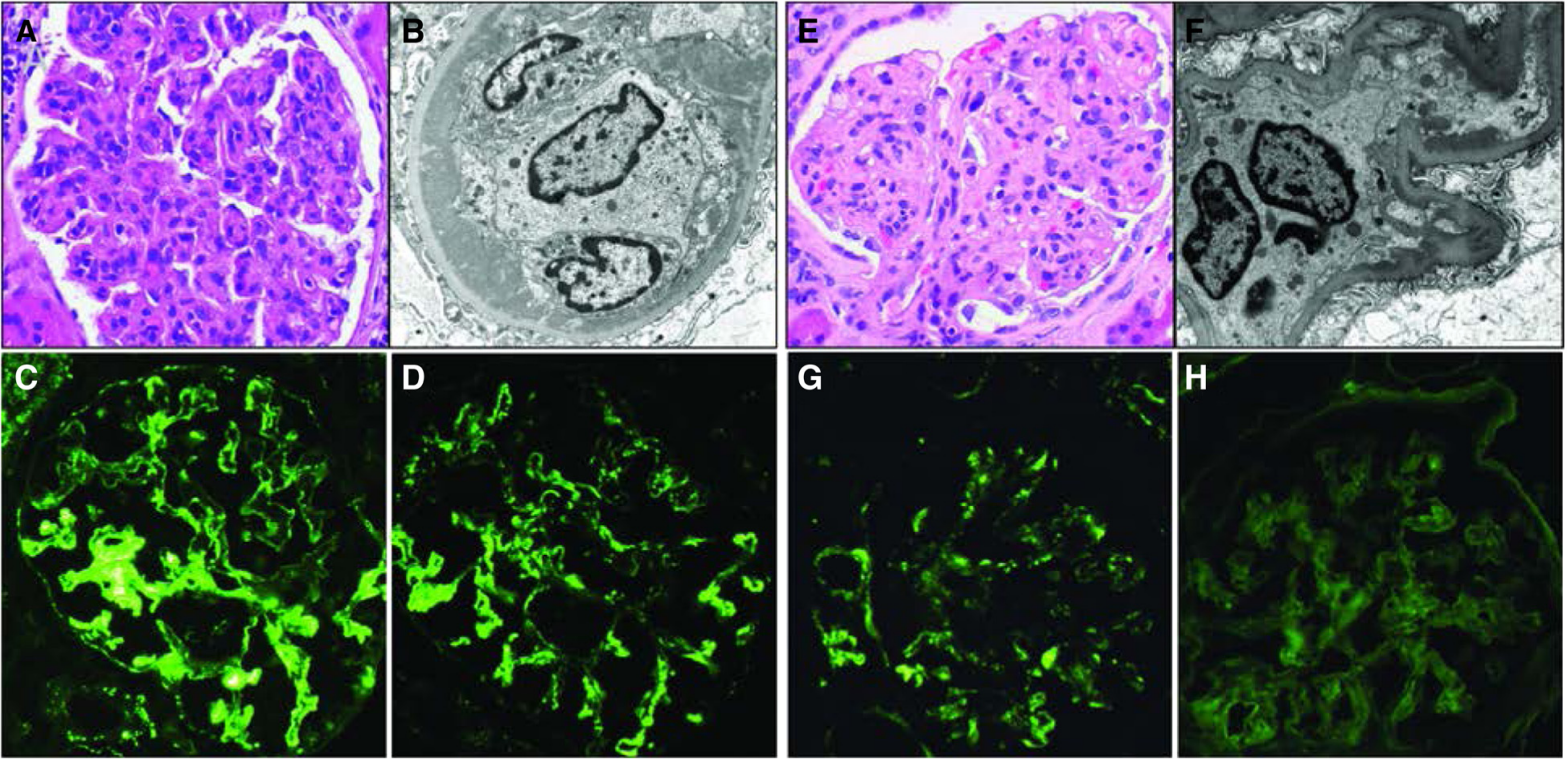
Histological changes of MPGN in kidney transplant biopsies. Typical light microscopic (LM), electron microscopy (EM) and immunofluorescence (IF) finding in cases previously classified as MPGN. Panel on left demonstrates a case reclassified as ICGN with C3 abnormalities, including (**a**) the classic MPGN pattern glomerulonephritis on LM (hematoxylin and eosin ×400), (**b**) large subendothelial electron dense deposits on EM (×1250 K) and granular mesangial and capillary wall staining for both (**c**) IgG and (**d**) C3 on IF. The panel on the right shows a case reclassified as a C3 glomerulopathy, with (**e**) a similar MPGN pattern on light microscopy (hematoxylin and eosin ×400), (**f**) smaller subendothelial deposits on EM (×7100) and granular mesangial and capillary wall staining for (**g**) C3, but no significant staining for (**h**) IgG. EM images stained with lead citrate/ uranyl acetate. Immunofluorescence stains are FITC-conjugated goat anti-human IgG (MP Biomedical) and FITC-conjugated goat anti-human C3 (Kent) all at × 400

We reviewed allograft biopsies that showed post-transplant MPGN recurrence and reclassified the 18 recurrences using the new MPGN classification system (Fig. 1). Table 5 delineates the MPGN subtypes of all post-transplant recurrences. In one of the recurrences reclassified as an ICGN, the type of MPGN changed in subsequent biopsies to CGN. Notably, this switch was observed following treatment with plasmapheresis. Additionally, there was a case of ICGN type that recurred with IgA dominance, in contrast to the original disease, which was a classic ICGN with IgG dominance. More interestingly, there was a case whose original disease was CGN but the recurrence fits the criteria of an ICGN diagnosis.

**Table 5:** Reclassified based on immunofluorescence C3 and IgG findings

Recurrence type at time of diagnosis (new classification)	Number of cases	Recurrence type at time of diagnosis (old classification)
ICGN	15	Type I: 14, Type III:1
CGN	2	Type I: 1, Type II: 1
ICGN-IgA dominant	1	Type I

*ICGN* immune complex mediated glomerulonephritis, *CGN* Complement mediated glomerulonephritis

### Treatment and outcome of post-transplant recurrent MPGN

**Fig. 2:**
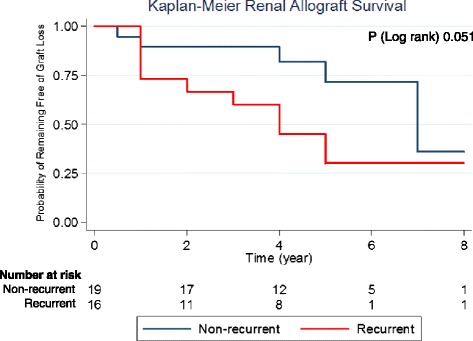
Kaplan Meier of allografts’ survival in patients with MPGN of ICGN type as original disease

Fourteen allografts out of the 18 recurrences received MPGN specific immunosuppressive therapy (Tables 6 & 7). Among the ICGN recurrences, immunosuppressive therapy included high-dose corticosteroids in 4 allografts, Rituximab in 5 allografts, plasma exchange alone in one allograft, plasma exchange with rituximab in 3 allografts. There are 2 cases of recurrence of CGN type and one of them was treated with eculizumab. One ICGN recurrence case was also found to have multiple myeloma and was treated with bortezomib. In 7 out of the 16 (43 %) transplants who developed post-transplant MPGN recurrence of ICGN type, the recurrence led to graft loss. In one of the two transplants who developed post-transplant MPGN recurrence of CGN type, the recurrence led to graft loss. The median time to graft loss after diagnosis in patients who lost their renal allografts was 6.5 months (range 2–18 months). Survival analysis among ICGN cases showed that overall renal allograft survival was not statistically different in both recurrent and non-recurrent groups although there was a trend of worse survival in the recurrent group (P log rank of 0.051) (Fig. 2).

**Table 6:** Variables associated with allograft loss among patients with MPGN ICGN-type recurrence after kidney transplantation by univariate Cox analysis (*n* = 16)

Independent variable	Hazard ratio (CI)	*P*-value
Age at transplantation	0.658 (0.326–1.815)	0.471
Gender (Male)	3.541 (0.325–11.785)	0.478
Race (Caucasian)	1.547 (0.354–7.548)	0.785
Allograft source (Living related)	1.547 (0.302–7.548)	0.914
Duration of dialysis	0.894 (0.541–1.325)	0.345
Preemptive transplantation	1.547 (0.458–5.879)	0.995
Previous failed transplantation	2.54 (00.485–9.356)	0.452
DGF (In deceased donor)	2.483 (0.4321–13.578)	0.546
Development of rejection	0.245 (0.008–3.024)	0.454
Use of ACEi/ARB	0.452 (0.081–0.952)	0.06
Low complement level at recurrence	4.201 (1.919–17.679)	0.022
Evidence of monoclonal gammopathy	3.054 (1.125–117.896)	0.45

*DGF* delayed graft function. *ACEi/ARB* angiotensin converting enzyme inhibitor/receptor/angiotensin receptor blocker

**Table 7:** Response of post-transplant MPGN recurrence to different treatments

Treatment	Number of allografts	Response to therapy^a^
High dose steroids	4	1
Rituximab ± plasmapheresis	8	3
Plasmapheresis	1	1
Eculizumab	1^b^	1
No change in therapy	4	3

^a^Response to therapy defined by improvement in GFR and no subsequent graft loss

^b^The case was CGN

Among cases of post-transplant MPGN recurrence, there was no statistically significant effect for age at transplantation, gender, race, allograft source, degree of mismatch, preemptive transplantation, severity of proteinuria at recurrence, development of rejection, complement level, or time to recurrence on graft loss (Table 6). However, the use of ACEi/ARB therapy was associated with a trend towards less graft loss (HR 0.301 and *P* = 0.07) that did not reach a statistical significance.

## Discussion

This study presents one of the largest case series of post-transplant MPGN recurrence in the literature and the first study to use the new MPGN classification system in assessing post-transplant MPGN recurrence. In this study, we demonstrated that post-transplant MPGN recurrence is quite common. We report a recurrence rate of 45 %. However, we do not routinely perform protocol post-transplant biopsies in our center and these data may underestimate the actual recurrence rate. In the study by Lorenz et al., the reported recurrence rate among MPGN type I patients is 41 % [8]. Moroni et al. reported a recurrence rate of 25 % among MPGN type I patients [12]. Green et al. reported a recurrence rate of only 19 %, and Braun et al. reported a recurrence rate among pediatric MPGN type II patients of 43 % [7, 11]. Thus the recurrence rate that we report is generally consistent with that reported in previous cohorts.

Consistent with previous reports, 55 % of MPGN recurrences were diagnosed within the first year of kidney transplantation. In the studies by Lorenz et al. and Green et al., all cases were diagnosed within 1.2 and 2.6 years of transplantation respectively [7, 8].

In our cohort, MPGN recurrence led to graft loss in half of the cases. This is consistent with the study by Moroni et al. in which graft loss occurred in 56 % of patients [12]. However, other studies showed different outcomes. In the study by Green et al., the recurrence led to graft loss in 88 %, as opposed to the observations by Lorenz et al., in which 16 % lost their grafts.

Our study analysis identified several factors associated with post-transplant MPGN recurrence. Lorenz et al. noted that living related transplantation may be associated with higher risk of recurrence but this did not reach statistical significance (*P* = 0.051) [8]. This observation was noted in the study by Green et al. as well, but this finding did not reach statistical significance either [7]. Our study demonstrated that living related donation is actually associated with higher risk of recurrence and this was statistically significant. This interesting association may be attributed to the possible common genetic predisposition in relatives of MPGN patients.

Interestingly, all preemptive transplants in our study developed MPGN recurrences, a significant difference with the balance of the cohort. This finding could be explained by the presence of continued underlying immune activity that would probably become suppressed by the immunosuppressive state of dialysis. However, we did not find an association between the duration of dialysis prior to transplantation and the development of recurrence. This contrasts with the findings of Green et al., wherein a trend toward association between shorter duration of dialysis before transplantation and recurrence was observed [7]. However, it is important to point out that our study remains a retrospective and a small study after all and we cannot ascertain these associations or prove causality.

In addition, our investigation confirmed the previous findings that low complement levels and the presence of monoclonal gammopathy are associated with higher risk of recurrence in ICGN cases [8, 12]. The association of recurrence with the presence of monoclonal gammopathy suggests that it may play a role in the pathogenesis of MPGN. One of our study patients who developed recurrence and had evidence of monoclonal gammopathy was later diagnosed with multiple myeloma and received bortezomib and rituximab. This patient maintained an excellent allograft outcome following treatment. The remainder of patients who had a recurrence of MPGN and evidence of MGUS in their serum and/or urine did not develop myeloma afterwards during the time of follow up.

In this series, patients who developed recurrence did not have worse allografts survival than those who did not, although graft loss after recurrence was high. It is pivotal to point out that the failure to demonstrate a difference in the outcome could be related to the small sample size. Furthermore, our study illustrates that clinically significant post-transplant MPGN recurrence responds poorly to immunosuppressive therapy. Less than half of the patients who received treatment in the form of high dose steroids, rituximab and/or plasmapheresis, or eculizumab responded to therapy and maintained their renal allografts. Interestingly, our study demonstrates the possible benefit of ACEi or ARB therapy in preventing graft loss. However, this should be interpreted carefully as patients who received such therapy are those who had stable renal function.

With regards to MPGN reclassification, most of the native and recurrent MPGN cases were reclassified to ICGN. This was expected because overall CGN is less common than ICGN. However, we observed a few cases in which the MPGN class changed over time. One patient whose original disease was CGN (confirmed by two biopsies) developed a recurrence after two years of transplantation that was reclassified as ICGN based on two separate biopsies several months apart. Another patient whose original disease was an ICGN (also confirmed by two biopsies) developed a recurrence of the CGN type. In these cases, it is difficult to distinguish whether this is a change in disease pattern or a development of de novo MPGN of different subtype. Based on the old classification, pre- and post- transplant biopsies were labeled as MPGN type I in both of the cases. More interestingly, there was one case with ICGN original disease that developed an MPGN recurrence. The first biopsy that showed recurrence was reclassified as ICGN but a subsequent biopsy five months later was reclassified to CGN. The change of type was noted after treatment with plasmapheresis. These findings shed the light on the possible change in microscopic findings of MPGN over time. This might be a problematic when deciding to treat this condition since the choice of treatment is dependent on the MPGN type.

There are some limitations to our study. It is a retrospective cohort study; hence there could be information bias due to missing data and we cannot prove causality in our findings. Although this is one of the largest studies that assessed post-transplant MPGN recurrence, it is a rare disease and the number of patients available for the study is small. Also, because the majority of our study population was type I and reclassified to ICGN, our study was not powered to detect differences in recurrence and outcome between the different types of MPGN. In other studies, the risk of recurrence in CGN was reported to be as high as 67 % [13].

## Conclusions

The positive findings of our study highlight several issues regarding kidney transplantation in ESRD patients due to MPGN. If these findings are confirmed by future prospective studies, extra effort should be made to look for living non-related donors. In the case of living related donors, a close surveillance is required after transplanting patients from living related donors. The same applies in the case of preemptive transplantation. These patients, along with patients with low complement level, should be intensively monitored for signs of disease recurrence or the transplant should be deferred to a time when recurrence is less likely. Our study confirmed the previously noted association of monoclonal gammopathy with MPGN recurrence. This finding should alert the transplant teams to perform screening for monoclonal gammopathy in MPGN patients undergoing evaluation for kidney transplantation. A referral to hematology may be required and close monitoring after transplantation should be employed in these cases. We should counsel MPGN patients undergoing kidney transplantation that this disease commonly recurs, and in many cases recurrence leads to graft loss. We should also be alert for the possibility of change of pathological findings of MPGN over time, which may alter our decision on treatment, and more frequent follow up biopsies may be warranted.

## Abbreviations

ACEi: Angiotensin coverting enzyme inhibitors
ARB: Angiotensin receptor blockers
ATN: Acute tubular injury
CGN: Complement-mediated glomerulonephritis
DGF: Delayed graft function
eGFR: estimated glomerular filtration rate
EM: Electron microscopy
ESRD: End stage renal disease
HLA: Human leukocyte antigen
HR: Hazard ratio
ICGN: Immune complex mediated glomerulonephritis
MPGN: Membranoproliferative glomerulonephritis

## References

[1] Swaminathan S, Leung N, Lager DJ, Melton LJ, Bergstralh EJ, Rohlinger A (2006). Changing incidence of glomerular disease in Olmsted County, Minnesota: a 30-year renal biopsy study. Clin J Am Soc Nephrol CJASN..

[2] Zhou F, Zhao M, Zou W, Liu G, Wang H (2009). The changing spectrum of primary glomerular diseases within 15 years: a survey of 3331 patients in a single Chinese centre. Nephrol Dial Transplant Off Publ Eur Dial Transpl Assoc - Eur Ren Assoc..

[3] Sethi S, Fervenza FC (2012). Membranoproliferative glomerulonephritis--a new look at an old entity. N Engl J Med..

[4] Cameron JS, Turner DR, Heaton J, Williams DG, Ogg CS, Chantler C (1983). Idiopathic mesangiocapillary glomerulonephritis. Comparison of types I and II in children and adults and long-term prognosis. Am J Med.

[5] Kumar V, Abbas A, Fausto N, Aster J. Pathologic basis of disease. 8th ed. Elsevier Saunders; 2009.

[6] Rennke HG (1995). Secondary membranoproliferative glomerulonephritis. Kidney Int..

[7] Green H, Rahamimov R, Rozen-Zvi B, Pertzov B, Tobar A, Lichtenberg S (2015). Recurrent membranoproliferative glomerulonephritis type I after kidney transplantation: a 17–year single-center experience. Transplantation..

[8] Lorenz EC, Sethi S, Leung N, Dispenzieri A, Fervenza FC, Cosio FG (2010). Recurrent membranoproliferative glomerulonephritis after kidney transplantation. Kidney Int..

[9] Andresdottir MB, Assmann KJ, Hoitsma AJ, Koene RA, Wetzels JF (1997). Recurrence of type I membranoproliferative glomerulonephritis after renal transplantation: analysis of the incidence, risk factors, and impact on graft survival. Transplantation..

[10] Appel GB, Cook HT, Hageman G, Jennette JC, Kashgarian M, Kirschfink M (2005). Membranoproliferative glomerulonephritis type II (dense deposit disease): an update. J Am Soc Nephrol JASN..

[11] Braun MC, Stablein DM, Hamiwka LA, Bell L, Bartosh SM, Strife CF (2005). Recurrence of membranoproliferative glomerulonephritis type II in renal allografts: the North American pediatric renal transplant cooperative study experience. J Am Soc Nephrol JASN..

[12] Moroni G, Casati C, Quaglini S, Gallelli B, Banfi G, Montagnino G (2011). Membranoproliferative glomerulonephritis type I in renal transplantation patients: a single-center study of a cohort of 68 renal transplants followed up for 11 years. Transplantation..

[13] Zand L, Lorenz EC, Cosio FG, Fervenza FC, Nasr SH, Gandhi MJ (2014). Clinical findings, pathology, and outcomes of C3GN after kidney transplantation. J Am Soc Nephrol.

